# Public health and nuclear winter: addressing a catastrophic threat

**DOI:** 10.1057/s41271-023-00416-7

**Published:** 2023-06-15

**Authors:** Andreas Vilhelmsson, Seth D. Baum

**Affiliations:** 1 Division of Occupational and Environmental Medicine, Lund University, Medicon Village (Building 402a), Scheelevägen 8, SE-223 81 Lund, Sweden; 2 Global Catastrophic Risk Institute, PO Box 40364, Washington, DC 20016 USA

**Keywords:** Global catastrophic risk, Public health, Nuclear winter, Climate change, Environment

## Abstract

Despite the end of the Cold War, the world still has thousands of nuclear weapons and adversarial relations between the countries that possess them. A nuclear war could cause large and abrupt global environmental change known as nuclear winter, with potentially devastating public health consequences. A significant line of natural science research characterizes nuclear winter and its potential effect on global food security, but less has been done on the human impacts and policy implications. Therefore, this Viewpoint proposes an interdisciplinary research and policy agenda to understand and address the public health implications of nuclear winter. Public health research can apply existing tools developed for the study of other environmental and military issues. Public health policy institutions can help build preparedness and community resilience to nuclear winter. Given the extreme potential severity of nuclear winter, it should be treated as a major global public health challenge to be addressed by public health institutions and researchers.

## Key messages

- A nuclear war could cause large and abrupt global environmental change.
- An interdisciplinary research and policy agenda to understand and address the public health implications of nuclear winter is needed.
- Public health policy institutions, like the WHO can help build preparedness and community resilience to nuclear winter.
- Nuclear winter should be treated as a major global public health challenge.

## Introduction

Public health has a long history of engagement of issues concerning nuclear war and nuclear weapons in general. Reports by the World Health Assembly in 1983 [1] and the World Health Organization (WHO) in 1984 [2] studied the local health effects of nuclear explosions and the role of health workers in issues of war and peace. Similar work has continued in the ensuing decades [3, 4]. A common theme is that the local effects of nuclear explosions are so extreme that public health response may not be feasible.

The local effects are undoubtedly important. However, the global environmental effects of nuclear war, commonly known as nuclear winter, could be significantly more severe, with effects including threats to global food security [5]. Indeed, nuclear winter may be one of the most far-reaching public health crisis scenarios. Furthermore, whereas there may be no surviving a direct hit from a nuclear weapon, there is significant potential to survive nuclear winter, especially if preparations are made in advance. Public health institutions and researchers have important roles to play in understanding the human implications of nuclear winter and leading emergency preparedness and response.

Despite the importance of nuclear winter, it is not prominent on the public health agenda of institutions or policy makers. There are some exceptions, such as a dedicated report on nuclear winter by the International Physicians for the Prevention of Nuclear War [6] and some discussion of nuclear winter by the International Committee of the Red Cross as part of a broader discussion of nuclear weapons risk and policy [4]. In contrast, for example, the only mentions of nuclear winter on the WHO website are in archives of documents from the 1980s. Its most recent report on nuclear weapons, “Health and environmental effects of nuclear weapons” [3], has not been updated since 1995, despite publishing a major report in 1987 giving attention to the climatic and nutritional impacts of nuclear war acknowledging the research on nuclear winter [7]. The lack of public health attention to nuclear winter is unfortunate because of the important ways that public health experts and institutions can contribute to understanding and mitigating the human toll of nuclear winter.

In this Viewpoint, we argue that public health experts (including researchers, advocates, and practitioners) and institutions (including the WHO, national public health agencies, and academia) should recognize nuclear winter as an important issue for the field to address. To accomplish this, a twofold agenda is needed. First, public health research is needed to help assess the effects of nuclear winter to human populations. This research should be both qualitative, characterizing the types of human impacts that nuclear winter may have, and quantitative, evaluating the overall severity of nuclear winter to inform public policy and other decision-making. Second, public health policy is needed to prepare for nuclear winter, and in the event that it would occur, help human populations survive. Some policy activities may need to wait until the research has progressed, but meanwhile there is much that can be done.

Given the global scope of nuclear winter, there should be participation from public health experts and institutions from around the world. Most nations do not possess nuclear weapons and are unlikely to be targeted in a nuclear attack. The nations that are targeted may have their public health infrastructure crippled by the local effects of nuclear explosions. Therefore, the nations that are not targeted may be especially capable of managing the public health effects of nuclear winter.

## The science of nuclear winter

The basic science of nuclear winter is as follows. Nuclear explosions on cities and industrial areas start large fires that may send smoke into the stratosphere, which is the second layer of the atmosphere above the clouds. There is no rain to wash the smoke out of the stratosphere and limited mixing between the stratosphere and the lower layer (the troposphere), so the smoke stays aloft for multiple years. While aloft, the smoke spreads around the globe and blocks incoming sunlight that reduces temperatures at the surface, hence the name nuclear winter [8].

Nuclear winter can be defined as global cooling from nuclear war so severe that winter temperatures occur during summer [9]. A more general definition, which we use here, also includes more moderate temperature declines and other environmental effects such as reduced precipitation and atmospheric ozone loss causing increase surface incidence of ultraviolet radiation [10]. Scientific research continues to clarify what the environmental effects of nuclear war would be. The intensity of the effects also depends on the war itself: larger nuclear wars cause more intense environmental change [5].

The environmental science of nuclear winter has been developed over several decades. Early research in the 1980s demonstrated that, under plausible nuclear war scenarios, the effect could be catastrophic to humans and ecosystems [9]. Recent research has used advanced climate models developed for the study of global warming, again finding catastrophic environmental change even for relatively small nuclear war scenarios [11]. An adjacent line of research has used crop models to estimate the implications for food security, finding a massive effect on a scale large enough to potentially cause global famine [5, 12]. Research on the human consequences of nuclear winter is more limited [4–6, 12]. Public health research can help address this gap, as we detail below.

It is not known if or when a nuclear war will occur, how large the war would be in terms of the number of nuclear warheads detonated, and what the environmental and human effects would be. Nuclear war is a quintessential example of a low-probability, high-severity risk. Only one nuclear war has occurred: World War II, specifically the bombings of Hiroshima and Nagasaki. Despite the absence of more recent nuclear wars, the ongoing probability of nuclear war is not zero. The potential for events to take a turn for the worse is demonstrated by the ongoing Russian invasion of Ukraine, which experts and political leaders have warned could escalate to nuclear war [13]. The exact size of the probability of nuclear war is not well-quantified and expert opinions on it vary [14]. For example, an important source of information is historical “near-miss” events such as the Cuban missile crisis; international security experts diverge on how “near” these events came to an actual nuclear war [15, 16].

Out of all the myriad harms from nuclear war, nuclear winter may be the most severe. The direct local effects from nuclear detonations can be quite large in their own right, including major medical harms [6]. The local effects clearly merit attention. However, nuclear winter stands out in its potential scale. It threatens catastrophic harm worldwide, even across regions that were not party to the war. In our globally connected world, secondary impacts would soon cascade across other systems like transport, food, water, trade, energy, finance, and communication and thus disrupting public health efforts.

Billions of lives may be at risk [5]. A collapse of the human civilization as we know it may be possible [10], with enormous implications for future generations [17]. However, the severity of nuclear winter is deeply uncertain, for two reasons. First, the nuclear war itself could be of any size from a single warhead to the detonation of all sides’ entire nuclear arsenals. The extent of escalation following an initial nuclear attack is “a giant unknown” [18]. Smaller nuclear war scenarios would generate relatively endurable nuclear winter. Second, human populations could respond to nuclear winter in a wide range of fashions, some more constructive than others. Nuclear winter has never previously occurred, so the tendency of human populations to endure it is unknown. The human toll of nuclear winter would likely also vary across different locations. For example, certain Southern Hemisphere island nations such as New Zealand may fare relatively well due to the effects of nuclear winter being less pronounced in addition to social and geographic advantages [19]. How populations would fare in a nuclear winter scenario may further depend on their degree of preparedness, including public health preparedness.

Therefore, while public health attention is warranted for the full range of nuclear war impacts [20], nuclear winter commands a special importance. Obviously, the best solution would be to prevent a nuclear war from ever happening and thus preventing a nuclear winter as well. However, given that global nuclear arsenals are expected to grow in the next decade as a response to increasing tensions between states [21], prevention should not be counted on as the only solution. Instead, the world should aim for multiple “lines of defense” to prevent, respond to, ensure resilience and to recover if things should go poorly [22].

## Research on the public health consequences of nuclear winter

Exactly how severe nuclear winter would be to human populations remains deeply uncertain. As noted above, prior research on nuclear winter has concentrated on environmental and agricultural dimensions, with relatively little on the human side, despite the potential for human and public health consequences to be catastrophic. A clearer accounting of the larger human consequences of nuclear winter is needed to evaluate how important of a policy priority it should be and what specifically should be done about it.

Fortunately, public health research can help with this. Public health research is highly interdisciplinary, with core disciplines including biostatistics, epidemiology, health policy and management, social and behavioral sciences, and environmental health sciences. This intellectual breadth is valuable for addressing the complex, multifaceted nature of nuclear winter. Furthermore, public health research is already accustomed to studying complex global challenges, including those with significant environmental components and even those with a military dimension. The study of nuclear winter would benefit significantly from public health contributions.

Nuclear winter would be an unusually difficult topic for public health research. It is highly complex, and it is also unprecedented in modern times. Some insight can be obtained from abrupt cooling events caused by volcanic eruptions [23], but no comparable cooling event has occurred since the advent of global public health systems. Therefore, typical data driven research approaches would not work. However, progress could still be made.

A starting point can be found in public health research on the environmental impact from global warming [24, 25]. This research assesses how environmental change can threaten the essential ingredients of good health: clean air, safe drinking water, nutritious food supply, and safe shelter. Similar research may be able to do the same for nuclear winter.

Several aspects of nuclear winter would benefit from public health research attention. These include increased ultraviolet radiation, secondary effects of diseases following destroyed sanitary facilities and contaminated water, and socioeconomic effects including social tensions and conflicts. These are concepts already familiar to public health. Additionally, it has been suggested that nuclear winter could result in the spread of infectious diseases, like plague, typhus, malaria, dysentery, and cholera [26, 27]*.* Given that infectious disease outbreaks can themselves be very severe, the possibility of outbreaks during nuclear winter is an important scenario to analyze.

We propose the following as promising directions for public health research on nuclear winter:

- *Risk assessment.* The World Bank commissioned the Global Burden of Diseases, Injuries, and Risk Factors Study (GBD) in the 1990s [28], thus providing a systematic scientific assessment on incidence, prevalence, and mortality for a mutually exclusive and collectively exhaustive list of diseases and injuries. The GBD is already used to calculate the global burden for war and armed conflict [29] and could be adapted to study public health effects from future nuclear winter scenarios, by integrating with climate and crop models.
- *Epidemiological forecasting*. Public health has extensive experience producing large epidemiological forecasts using statistical and computational methods to predict when and where disease outbreaks may occur [30]. This research has been a valuable input to policy and other decision-making for influenza [31]. Data on nuclear winter may be scarce, but insight could be obtained adapting prior research on other environmental threats such as climate change [32] and pollution [33].
- *Attribution science*. Public health researchers have made substantial progress on characterizing the health burdens attributable to global warming [33], although it is more difficult to accurately estimate the scale and nature of the impacts. The same can be said for nuclear winter. Indeed, health impacts that are potentially attributable to global warming, such as infectious disease, foodborne disease, and malnutrition, may also result from nuclear winter. Likewise, the models developed for global warming attribution studies could be adapted for nuclear winter.
- *Geographic analysis*. Geographic information system (GIS) is an important research tool to understand the geographic distribution of public health issues, for instance where diseases are found and how they relate to the environment. At the same time, it functions like a communication tool to illuminate how human–environment interactions can affect human health [34]. Nuclear winter has substantial geographic heterogeneity deriving from the particularities of nuclear war scenarios (which countries would be targeted), air circulation in the stratosphere, agricultural geography, and other phenomena. Integration of geographic information about nuclear winter and public health could generate valuable research results.

## Public health policy to address to nuclear winter

As a major catastrophic threat, nuclear winter must be addressed by policy makers to ensure the shared future of humanity. However, as a multifaceted, cross-cutting issue, nuclear winter does not fit neatly within any one type of policy institution. It is not exactly a defense issue, or an environmental issue, or a health issue. Therefore, it gets less policy attention than it deserves.

Prior research has proposed a variety of policy responses to nuclear winter. These include nuclear disarmament [35], adjusting the composition of military forces used for deterrence [10], agricultural measures to address food security challenges [36], and reducing the probability of nuclear war [37]. A public health policy emphasizing preparedness for nuclear winter would complement these other measures by improving outcomes for human populations that also could be used for other catastrophic threats, not least being climate change. Preparedness for nuclear winter can include developing stockpiles for food, water, vaccines, and other medicines and other resources necessary for populations to survive until environmental conditions improve. During nuclear winter, such resources could be the difference between life and death for a large portion of the human population.

The human element is critical to nuclear winter preparedness. Any resources prepared in advance would be of little value of people could not successfully use them during the event. Nuclear winter would likely pose extreme challenges for emergency response, including acute shortages of staff, materials, and time. Therefore, preparedness should include developing the capacities and skills that communities would need to in order to be able to implement emergency response.

A public health approach to capacity building can enable societies to develop the skills and enabling systems necessary to survive catastrophic events [38]. This type of intervention aims to improve the practice of public health practitioners and the infrastructure of public health organizations by enhancing and sustaining individual and organizational capacity to address local health issues [39]. Public health institutions could also play an important part in helping organize nonmilitary plans that prepare communities and civilians for a military attack, such as was done during the Cold War, by for instance establishing Civil Defense Emergency Hospitals in rural areas less likely to be hit [40]. Nuclear winter poses distinct challenges that will need dedicated human capacity to address.

Building community resilience, meaning a community’s capability to rebound from a disaster, is a cornerstone of public health emergency preparedness. Modern public health research demonstrates the importance of human capacity in responding to a wide range of disaster scenarios [41].

For that reason, we call for nuclear winter to be treated as a public health issue to be addressed by public health institutions and researchers. The activities described here are firmly within the capacity of public health and are not already being performed by other institutions or researchers to any significant extent, although this may look different in different parts of the world. The public health community has an important contribution to make to addressing nuclear winter.

Finally, public health institutions and researchers can contribute to broader policy conversations on nuclear weapons, such as regarding arms control and the pursuit of peace between nuclear-armed countries. These policies reduce the entire risk of nuclear war, including but not limited to the risk of nuclear winter. Some research has called for public health to support nuclear disarmament, including via the recent Treaty on the Prohibition of Nuclear Weapons (TPNW) [42], which has received institutional public health support [43], though the merits of the TPNW are not straightforward in relation to states’ security concerns and other disarmament efforts [44].

As the public health arm of the United Nations, the WHO should play a leading role. While it is encouraging that the United States National Academy of Sciences, Engineering, and Medicine is currently undertaking a Congressionally mandated study of the environmental effects of nuclear war [45], the WHO are uniquely positioned to address a global health issue of this magnitude that also can coordinate the work with National Public Health Institutes (NPHIs) and other relevant agencies within the UN system. Specifically, the WHO should reintroduce the topic of nuclear weapons to its ongoing work and explicitly include nuclear winter. This work should include a new guiding report to set the initiative for other public health work on nuclear winter. The work should also include regular updates to account for new developments in nuclear winter science and policy together with NPHIs and Academia.

## Conclusion

Nuclear winter is one of the largest threats to global public health. Nuclear war can occur at any time, and nuclear winter may soon follow, leaving human populations worldwide in desperate conditions. How successfully populations cope under these conditions may depend on how effectively they have prepared. Nuclear winter is a multifaceted threat, and multifaceted preparations are needed. Public health can and should make major contributions to addressing nuclear winter, including both research and policy, just like pandemic preparedness is organized and monitored on a global scale.

Although there are synergies to be made with research on public health dimensions of armed conflict [29] environment, and global warming [24], addressing nuclear winter will require some dedicated attention. How scarce public health resources should be allocated across nuclear winter and other issues is beyond the scope of this paper. The current public health allocation to nuclear winter is approximately zero. Given the catastrophic potential of nuclear winter, more is clearly needed. As long as some states continue to possess nuclear weapons or have the capacity to produce them, societies must be prepared to handle a potential future impact.

We simply cannot afford not to.
